# Clinical History, Spirometry, and CT Features Can Predict Dyspnea in Smokers with and without Spirometry-Defined COPD

**DOI:** 10.1007/s00408-026-00871-5

**Published:** 2026-02-19

**Authors:** Joosun Shin, Mary E. Cooley, Marilyn J. Hammer, Chi-Fu J. Yang, Uno Hajime, Enrico Maiorino, Richard Casaburi, Adel R. El Boueiz, Raúl San José Estepar, Peter J. Castaldi

**Affiliations:** 1 Phyllis F. Cantor Center for Research in Nursing and Patient Service, Dana-Farber Cancer Institute, Boston, MA USA; 2 Channing Division of Network Medicine, Brigham and Women’s Hospital, Boston, MA USA; 3 Division of General Internal Medicine and Primary Care, Brigham and Women’s Hospital, Boston, MA USA; 4 Division of Population Sciences, Dana-Farber Cancer Institute, Boston, MA USA; 5 Division of Thoracic Surgery, Department of Surgery, Massachusetts General Hospital, Boston, MA USA; 6 Applied Chest Imaging Laboratory, Department of Radiology, Brigham and Women’s Hospital, Boston, MA USA; 7 Respiratory Research Center, Los Angeles Medical Center, Lundquist Institute for Biomedical Innovation at Harbor-University of California, Torrance, CA USA; 8 Division of Pulmonary and Critical Care Medicine, Brigham and Women’s Hospital, Harvard Medical School, Boston, MA USA; 9 Harvard Medical School, Boston, MA USA; 10 Joe C. Wen School of Nursing, University of California, Los Angeles, CA USA

**Keywords:** Machine learning, Spirometry, Quantitative chest computed tomography

## Abstract

**Rationale:**

Dyspnea is common in smokers with or without chronic obstructive pulmonary disease. Its multifactorial nature makes it challenging to identify specific factors causing dyspnea in smokers with and without chronic obstructive pulmonary disease.

**Objectives:**

The study aims to identify associations between clinical history, spirometry, and computed tomography findings related to dyspnea in smokers, and to develop and compare dyspnea models using different variable combinations.

**Methods:**

Dyspnea was defined as a self-reported modified Medical Research Council dyspnea scale score ≥ 2. Participants from the COPDGene Study dataset were utilized and split into training and testing samples (80%/20%) to develop and validate a predictive model. The ECLIPSE Study was used for external validation. Bivariable and multivariable logistic regression analyses were used to examine factors associated with dyspnea. Predictive models were developed using Elastic Net.

**Main Results:**

The final prediction model demonstrated good predictive performance, achieving an area under the curve of 0.85 in the test set and 0.80 in the external dataset. We confirmed prior associations with dyspnea and identified novel interactions of multiple risk factors with chronic obstructive pulmonary disease severity.

**Conclusions:**

Dyspnea in smokers with and without chronic obstructive pulmonary disease can be predicted with high accuracy using a model that utilizes clinical history, spirometry, and chest CT imaging. To make accurate predictions, data from at least two of the three variable domains (clinical history, spirometry, or chest CT imaging) was required.

**Supplementary Information:**

The online version contains supplementary material available at 10.1007/s00408-026-00871-5.

## Highlights

- Interaction models indicate that while chronic bronchitis and respiratory exacerbations have a greater impact on dyspnea in smokers with preserved spirometry compared to chronic obstructive pulmonary disease (COPD) patients, computed tomography (CT) emphysema and systemic inflammation are more pronounced in smokers with Global Initiative for COPD (GOLD) stages 2–4 COPD.
- Elastic Net models accurately predict dyspnea using clinical data, spirometry, and chest CT.
- To make accurate dyspnea predictions, data from at least 2 of the 3 domains (clinical history, spirometry, or chest CT) were required.

**Supplementary Information:**

The online version contains supplementary material available at 10.1007/s00408-026-00871-5.

## Introduction

Dyspnea is a prevalent and multifactorial symptom among smokers, both with and without chronic obstructive pulmonary disease (COPD). More than half of smokers experience dyspnea, which arises from a variety of risk factors, including lung and cardiovascular disease, obesity, anemia, and anxiety [1, 2]. Dyspnea may stem from multiple co-existing mechanisms. One significant clinical challenge is accurately identifying the strongest underlying factors associated with dyspnea, a task essential for optimizing diagnosis, prognosis, and management by identifying high-risk individuals in a timely manner. Traditional statistical regression models are limited in their ability to identify the most important factors that are concurrently contributing and interplaying in complex associations with the outcome.

Artificial intelligence/Machine learning (AI/ML) has emerged as a novel method for analyzing complex associations, offering benefits over traditional statistical methods when multiple, interrelated factors are involved. Previous AI/ML models for dyspnea have been developed primarily in general population samples or in patients with lung cancer, yielding area under the receiver operating curves (AUROCs) ranging from 0.55 to 0.81 [2–5]. Among these, the model proposed by Olsson et al. achieved the highest accuracy by integrating 449 variables spanning clinical history, spirometry, and CT imaging in a cohort of 28,730 smokers and non-smokers aged 50 to 64 years. [2] This work demonstrated that multimodal predictors could enhance dyspnea prediction. However, to date, no models have specifically targeted former and current smokers with spirometry-defined COPD, particularly Global Initiative for Chronic Obstructive Lung Disease (GOLD) stages 2–4, where symptom burden and disease complexity are most significant.

Dyspnea, a central component of COPD diagnosis and assessment [6], is closely linked to clinical history (e.g., demographics, comorbidities), spirometry, and quantitative chest computed tomography (CT) [7–12]. Building on this knowledge, we aimed to identify key determinants of dyspnea in a large cohort of former and current smokers enriched for spirometry-defined COPD, and to develop and evaluate predictive models that incorporate data from clinical history, spirometry, and quantitative CT (qCT) chest imaging. We hypothesized that each of these three domains captures distinct yet complementary risk factors for dyspnea that interact with the severity of spirometry-defined COPD to influence dyspnea presence. Given that CT imaging is not always readily available, we compared models based on different combinations of these predictors to determine which provides the most accurate and clinically meaningful prediction of dyspnea. Large datasets from the COPDGene study were used for model development, and the Evaluation of COPD Longitudinally to Identify Predictive Surrogate Endpoints (ECLIPSE) study for external validation.

## Methods

### Discovery and Validation Samples

The COPDGene Study is a 21-center, ongoing longitudinal observational study of non-Hispanic White (NHW) and African American (AA) individuals, most of whom had a > 10 pack-year smoking history [13]. Study subjects were initially enrolled from 2007 to 2011, and each subject received extensive lung phenotyping at five-year intervals, incorporating spirometry, qCT imaging phenotypes, and questionnaires. The current study used the 5-year follow-up (Phase 2) data, which included more comprehensive features linked to dyspnea, such as the self-reported Hospital Anxiety and Depression Scale. [14] The data used for this analysis included 5016 individuals aged 45–80 with > 10 pack-years of smoking history. The ECLIPSE study, which included 2290 subjects, the majority of whom were NHW aged 40–75 with a smoking history of > 10 pack-years in the United States (US) and Europe, was used for external validation.

### Feature Selection and Data Preprocessing

#### Response Variable

In this paper, the presence of dyspnea (yes/no) was the dependent variable for association analyses and predictive models. Former and current smokers with dyspnea were defined as those with a self-reported modified Medical Research Council (mMRC) dyspnea score of 2 or higher, based on previous literature that identified cutpoints [15, 16]. The mMRC dyspnea scale ranges from 0 (dyspnea only on strenuous exercise) to 4 (dyspnea when dressing or too dyspneic to leave the house) [16, 17].

#### Feature Selection

Feature selection occurred in two steps. Firstly, clinical domain knowledge and the literature guided the selection of features from three data types: clinical history, spirometry, and qCT imaging. Next, we excluded variables with over 20% missing values. Correlations between continuous variables (Pearson’s correlation) and categorical variables (Kendall’s rank correlation) were calculated (Supplemental Figure 1). For pairs of variables with correlations exceeding 0.8, only one was retained for analysis to minimize multicollinearity based on clinical relevance. Detailed information on the final set of variables and their definitions is listed in Supplemental Table 1.

#### Data Preprocessing

The COPDGene study dataset was randomly divided into a training and a test set. Eighty percent of the subjects comprised the training cohort for dyspnea prediction model development, and the remaining 20% comprised the test cohort for internal validation. The ECLIPSE dataset was used for external validation.

### Statistical Analysis

Bivariable and multivariable logistic regression analyses examined the association between the presence of dyspnea (yes/no) and clinical history, spirometry, and qCT imaging variables. In these models, the presence of dyspnea (yes/no) was the dependent variable. The clinical history, spirometry, and qCT imaging were included as independent variables. Continuous variables were mean-centered and scaled (divided by standard deviation) before regression analyses. Each variable was tested for association with dyspnea in separate (i.e., one model for each variable of interest) bivariable models. Each variable was also assessed in a separate multivariate model adjusting for GOLD spirometric stage [6], age, sex, and race. The variable “GOLD spirometric stage” was defined as spirometry-defined COPD severity as used by GOLD and coded into four groups for these analyses: normal spirometry (GOLD stage 0), preserved ratio impaired spirometry (PRISm), mild COPD (GOLD stage 1), and moderate to severe COPD (GOLD stages 2–4; collapsed into a single category). Smokers with normal spirometry were the reference group. Finally, multivariate regression analysis with interaction terms was conducted to test the potential interaction of spirometry-defined COPD severity used by GOLD with comorbidities, spirometry, and qCT imaging factors in predicting dyspnea.

#### Prediction Model Training

Elastic net regression was used to build the dyspnea predictive model using the ‘glmnet’ R package [18]. Alpha and lambda values were optimized entirely within the training set using the cross-validation procedure implemented in ‘glmnet’. The final models were fit exclusively on the training data using the optimal alpha and lambda value derived from the training set. Subsequently, to test for interactions between GOLD spirometric stage (COPD severity) and clinical history, spirometry, and chest CT imaging in relation to dyspnea, we developed a linear hierarchical pairwise-interaction model, known as the Group-Lasso INTERaction-Net (GLINTERNET) model, using the ‘glinternet’ R package [19]. The GLINTERNET model automatically selects and estimates main effects and pairwise interactions with COPD severity, using group lasso regularization while enforcing strong hierarchical constraints, ensuring that interactions are included only when their main effects are relevant. Additionally, we conducted stratified analyses based on COPD status to evaluate whether the model’s performance varied by creating three distinct elastic net prediction models: (1) all former and current smokers; (2) former and current smokers without COPD (GOLD stage 0); and (3) former and current smokers with moderate to very severe COPD (GOLD stages 2–4).

#### Model Comparisons with Different Variable Sets

To develop clinically relevant prediction models for dyspnea, we developed eleven models using different combinations of variables and tested their performance. Supplemental Table 1 shows the variable sets used.

#### Prediction Model Evaluation

For each model, we calculated the AUROC, and the Brier score and Spiegelhalter z tests were used to measure model calibration [20]. Pairwise comparison of model performance was performed using the DeLong test. Variable importance was assessed using the ‘varImp’ function in the Caret R package, which calculates variable importance scores based on the beta-coefficients from an elastic net model [21]. Then, we selected the top 10 continuous and categorical variables, respectively, and created variable importance plots. All analyses were conducted using R software (version 4.1.3). To ensure transparency and completeness in reporting, the study adhered to the TRIPOD guidelines [22]. The TRIPOD checklist (Supplemental Table 2) was used to guide the presentation of study objectives, data sources, statistical methods, and results.

## Results

### Subject Characteristics

**Fig. 1:**
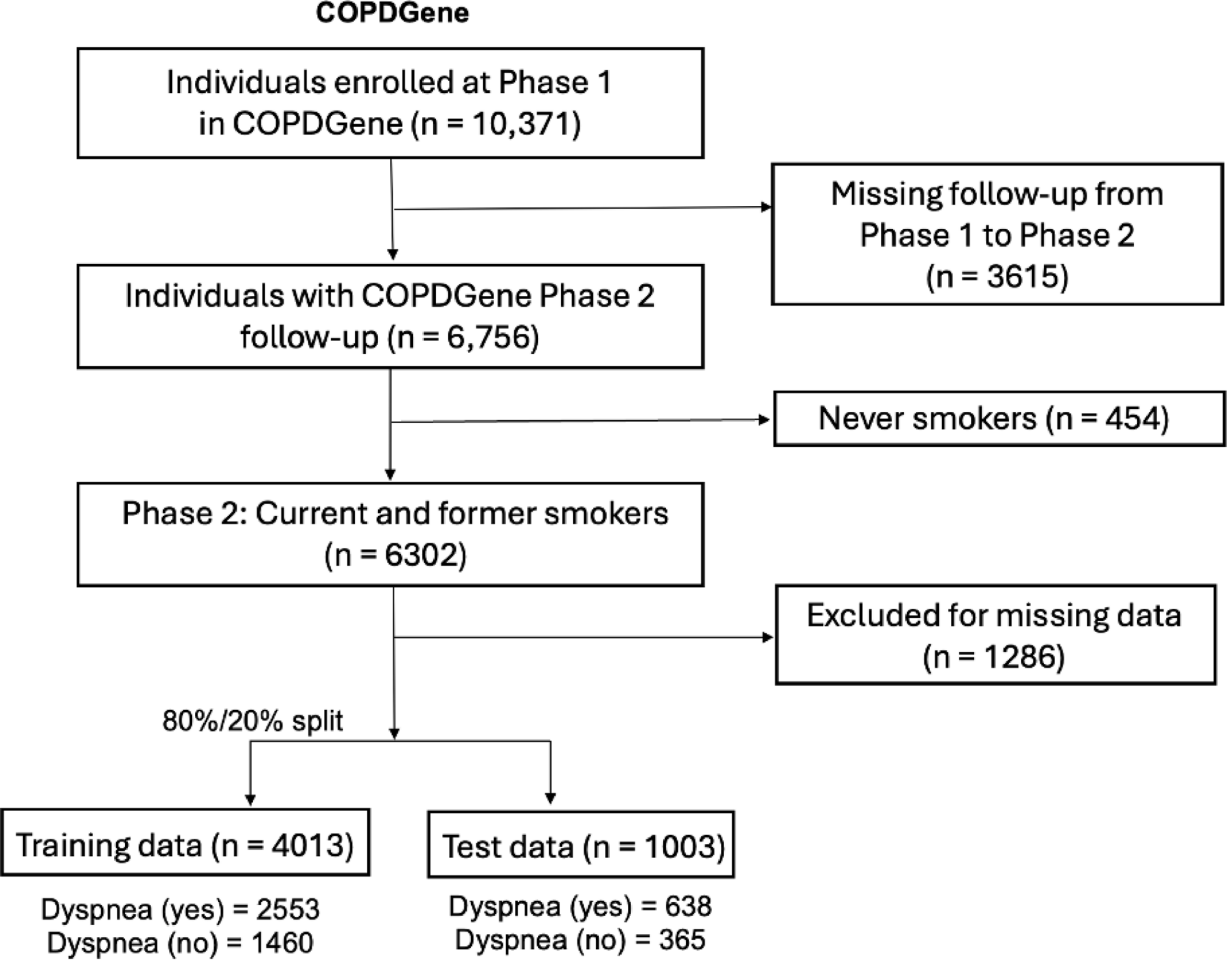
Study flow diagram

Figure 1 shows the study flowchart. The characteristics of the subjects in the COPDGene training dataset are shown in Supplemental Table 3. Out of 4,013 former and current smokers in the training dataset, 36.4% reported experiencing dyspnea.

### Variables with Significant Interactions with COPD Severity on Dyspnea

All 28 variables significantly associated with dyspnea in the multivariable analysis (Supplemental Table 4) above were also tested for interaction with GOLD spirometric stage. Interaction terms were included to evaluate whether spirometry-defined COPD severity used by GOLD affects the association of each candidate risk factor with dyspnea (Table 1). In terms of interactions with GOLD stage 2–4 COPD compared to smokers with normal spirometry, we observed that CT-quantified emphysema (interaction term b = 0.76, SE = 0.26, *p* < 0.005) and NLR (b = 0.20, SE = 0.08, *p* < 0.05) had a stronger effect in GOLD stages 2–4 COPD. On the other hand, multiple variables had a larger effect on dyspnea in smokers with normal spirometry, including chronic bronchitis (b =  − 0.73, SE = 0.22, *p* < 0.005) and frequent respiratory exacerbations (b =  − 0.99, SE = 0.34, *p* < 0.005). Regarding interactions with PRISm compared to smokers with normal spirometry, we observed that CT-quantified emphysema (interaction term b = 1.16, SE = 0.45, *p* < 0.05) had a more substantial effect in PRISm.

**Table 1:** Multivariable COPD interaction models of the relationship between dyspnea and clinical history, spirometry, and chest CT imaging characteristics

	Beta (SE)	GOLD stages 2–4 COPD Beta (SE)	GOLD stages 2–4 COPD x row variable Beta (SE) ^§^
CT Emphysema (LAA%950)	− 0.23 (0.25)^NS^	1.75 (0.14)^**^	0.76 (0.26)^**^
NLR	0.16 (0.06)^*^	1.86 (0.09)^**^	0.20 (0.08)^*^
Hypertension	0.51 (0.12)^**^	2.11 (0.13)^**^	− 0.38 (0.17)^*^
Osteoarthritis	0.76 (0.13)^**^	2.11 (0.10)^**^	− 0.58 (0.19)^**^
Rheumatoid arthritis	1.03 (0.20)^**^	2.00 (0.09)^**^	− 0.71 (0.34)^*^
Chronic bronchitis	1.28 (0.18)^**^	1.94 (0.10)^**^	− 0.73 (0.22)^**^
Frequent respiratory exacerbation	1.90 (0.29)^**^	1.88 (0.09)^**^	− 0.99 (0.34)^**^
Cognitive disorder	1.48 (0.39)^**^	1.95 (0.09)^**^	− 1.15 (0.53)^*^

	Beta (SE)	PRISm Beta (SE)	PRISm x row variable Beta (SE)^§^
CT Emphysema (LAA%950)	− 0.23 (0.25)^NS^	1.55 (0.23)^**^	1.16 (0.45)^*^

	Beta (SE)	GOLD stage 1 COPD Beta (SE)	GOLD stage 1 COPD x row variable Beta (SE)^§^
Smoking pack-years	0.32 (0.07)^**^	0.10 (0.15)^NS^	− 0.39 (0.14)^**^

COPD, chronic obstructive pulmonary disease; CT, computed tomography; GOLD, Global Initiative for COPD; NLR, neutrophil-to-lymphocyte ratio; LAA, low attenuation areas; PRISm, preserved ratio impaired spirometry; SE, standard error. ^NS^not significant; *p < .05; **p < .005. Each row corresponds to a separate multivariate interaction model. Smokers with normal spirometry were the reference group. ^§^The equation of multivariate COPD interaction models for dyspnea = dyspnea presence (yes/no) ~ COPD stage + age + sex + race + each candidate variable + COPD stage. *each candidate variable. COPD stage is coded as normal spirometry (reference), PRISm, GOLD 1, and GOLD 2–4.

### Dyspnea Prediction Models Using Different Sets of Variables

**Fig. 2:**
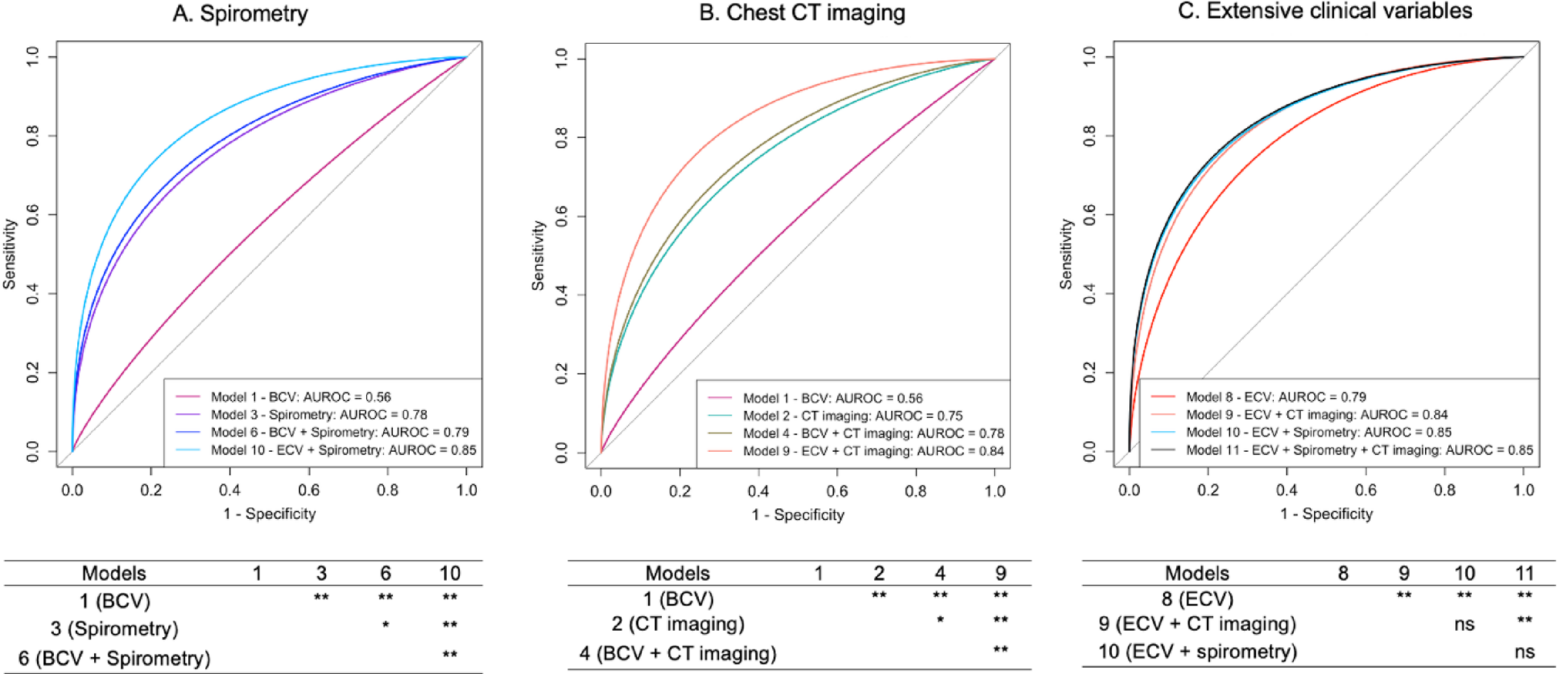
A–C The area under the receiver operating characteristic curves (AUROCs) for the elastic net prediction models with various variable combinations predicting dyspnea in the test dataset from the COPDGene study (Visit 2). **A** displays different combinations of clinical variables and spirometry. **B** presents various clinical variables and chest CT imaging combinations. **C** illustrates various combinations of extensive clinical, spirometric, and/or chest CT imaging variables. The tables summarize the pairwise DeLong *P* values of the model comparisons. Abbreviation: AUROC = area under the receiver operating characteristic curve; BCV = basic clinical variables; COPD = chronic obstructive pulmonary disease; CT = computed tomography; ECV = extensive clinical variables. Please refer to Supplemental Table 1 for a listing of BCV and ECV. **p < 0.005; *p < 0.05; ns = not significant

**Fig. 3:**
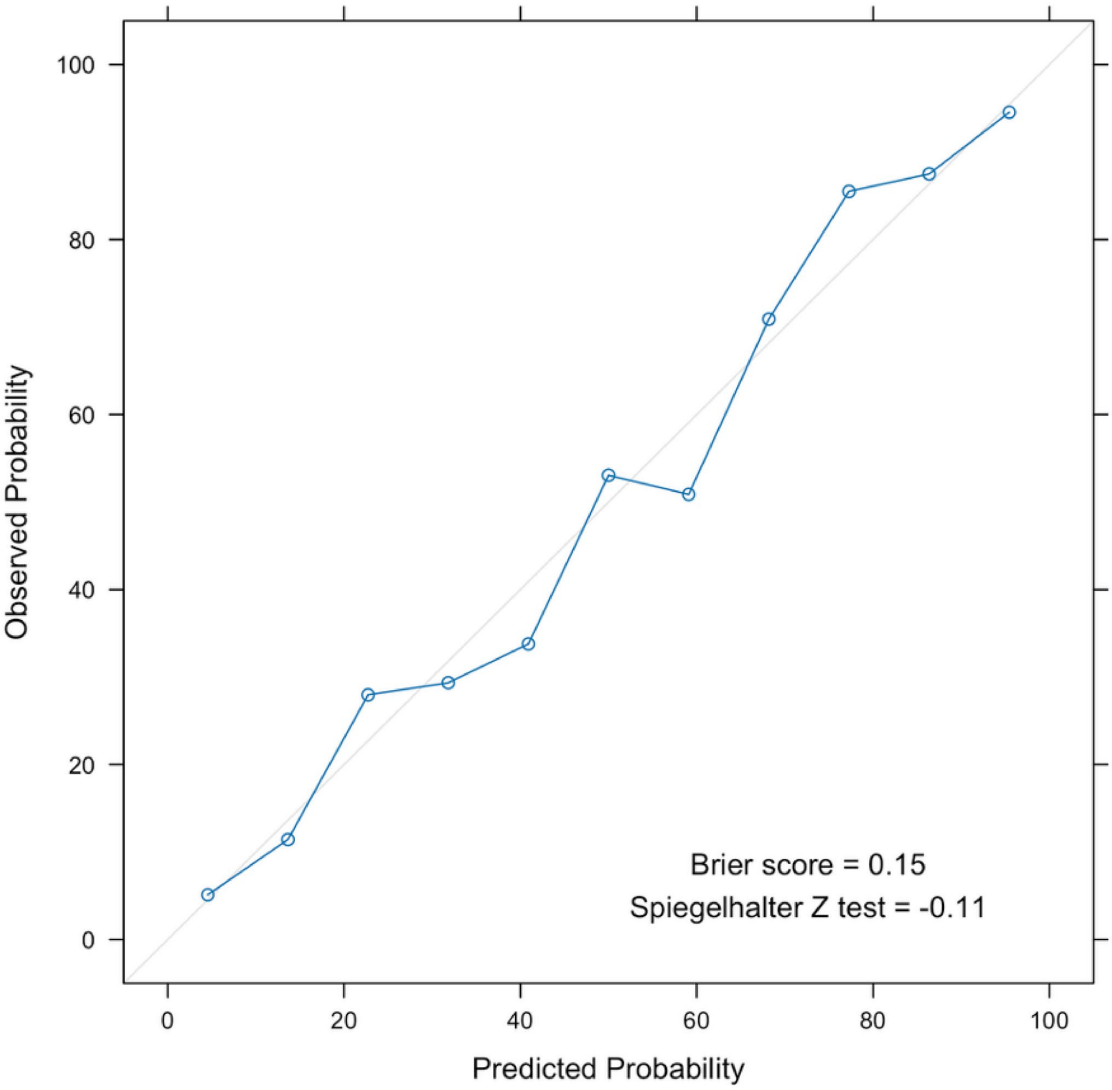
Calibration plot of model 11 in the test dataset of the COPDGene study (Visit 2). The calibration plots assess the agreement between predicted and observed probabilities of dyspnea occurrence. The Brier Score is 0.15, indicating that the final dyspnea model is well-calibrated. The Spiegelhalter Z statistic is − 0.11, which suggests no significant overestimation or underestimation of the probability of dyspnea occurrence. COPD = chronic obstructive pulmonary disease

We then evaluated the performance of prediction models for dyspnea, fitting models that used different combinations of clinical history, spirometry, and qCT imaging. The AUROCs for these models ranged from 0.56 to 0.85 (Table 2). Figure 2A–C illustrate these results. When comparing the three variable sets in isolation, models with spirometry, chest CT imaging, or extensive clinical variables alone had AUROCs of 0.75–0.79. Basic clinical variables alone (age, sex, race) yielded poor performance. For AUROC > 0.8, at least two variable sets needed to be combined, and the best performance was achieved with extensive clinical variables combined with either CT or spirometry. Among these, models 10 and 11, which included extensive clinical variables and spirometry (model 10) or extensive clinical variables, spirometry, and qCT imaging (model 11), achieved the best performance (AUROC = 0.847 and 0.850, respectively; DeLong p-value comparing model 10 and 11 = 0.34). We observed that this model was well-calibrated with a Brier score of 0.15 and a Spiegelhalter Z-statistic of − 0.11 (Fig. 3).

**Table 2:** Performance outcomes of variable sets to predict dyspnea in the test dataset in COPDGene visit 2

Model	Variable sets	AUROC (95% CI) in test set
1	Basic clinical variables ^†^	0.56 (0.52 – 0.60)
2	Chest CT imaging ^††^	0.75 (0.72 – 0.79)
3	Spirometry ^‡^	0.78 (0.74 – 0.81)
4	Basic clinical variables + chest CT imaging	0.78 (0.75 – 0.81)
5	Spirometry + chest CT imaging	0.79 (0.76 – 0.82)
6	Basic clinical variables + spirometry	0.79 (0.76 – 0.82)
7	Basic clinical variables + spirometry + chest CT imaging	0.81 (0.78 – 0.84)
8	Extensive clinical variables ^§^	0.79 (0.76 – 0.82)
9	Extensive clinical variables + chest CT imaging	0.84 (0.81 – 0.86)
10	Extensive clinical variables + spirometry	0.85 (0.82 – 0.87)
11	Extensive clinical variables + spirometry + chest CT imaging	0.85 (0.83 – 0.87)

AUROC, area under the receiver operating characteristic curve, 95% CI, 95% confidential interval; CT, computed tomography. ^†^Basic clinical variables = Age, race, and sex. ^††^Chest CT imaging = CT quantified total emphysema, %LAA-950; Emphysema distribution (Upper over lower lung third %LAA-950 ratio); Pi10 (Square root of the wall area of a hypothetical airway of 10-mm internal perimeter); Airway wall thickness (Obtained along the center line of the lumen, in the middle third of the airway segment, for one segmental airway of each lung lobe); CT-measured total lung volumes at end-inspiration. ^‡^Spirometry = Pre-bronchodilator FEV1 (L); Pre-bronchodilator FEV1/FVC, predicted; pre-and post-bronchodilator FEV1, % change. ^§^Extensive clinical variables = Age; Sex; Race; Body mass index (kg/m^2^); Smoking status (Former/current); Smoking pack-year history; Frequent respiratory exacerbation (Respiratory exacerbation > 2 per year); Heart rate (bpm); Pneumothorax (Self-report); Congestive heart failure (Self-report); Diabetes (Self-report); Hypertension (Self-report); Hyperlipidemia (Self-report); Pulmonary embolism or Deep vein thrombosis (Self-report); Peripheral vascular disease (Self-report); Vertebral compression fracture (Self-report); Hip fracture (Self-report); Osteoarthritis (Self-report); Osteoporosis (Self-report); Rheumatoid arthritis (Self-report); Chronic bronchitis (Chronic cough and phlegm for 3 months/year for at least 2 consecutive years); Cognitive disorder (Self-report); Anemia (Self-report); Kidney disease (Self-report); Liver disease (Self-report); Lung cancer (Self-report); Cardiovascular disease (A composite of self-reported diagnoses of angina, coronary artery disease, heart attack, or atrial fibrillation, or self-reported history of coronary artery bypass grafting or angioplasty); Cerebrovascular disease (A composite of self-reported diagnoses of stroke or transient ischemic aneurysm); Gastrointestinal disease (A composite of self-reported diagnoses of gastroesophageal reflux disease or stomach ulcer); Depression (Hospital Anxiety and Depression Scale-Depression > 7); Anxiety (Hospital Anxiety and Depression Scale-Anxiety > 7); Hemoglobin (g/dL); Eosinophil (k/uL); Neutrophil-to-lymphocyte ratio (NLR)

To determine whether including interaction terms was useful, we also trained a model with pairwise interaction terms for COPD severity using the GLINTERNET method. The final model identified that the presence of anxiety interacts with COPD severity in predicting dyspnea (interaction term b =  − 0.02). AUROC in a test set of subjects was 0.850. Since this GLINTERNET model did not perform better than model 11 (DeLong *p*-value = 0.99), [23] subsequent investigations focused only on Elastic net regression models without interaction terms.

Additionally, stratified analyses were conducted to evaluate whether models trained on subjects with COPD alone or on subjects with normal spirometry alone might perform better than models trained on all subjects. For smokers with normal spirometry, the AUROC in a test set of subjects with normal spirometry was 0.76 (AUROC for model 11 in these data was 0.85, Delong *p*-value = 0.004). For smokers with GOLD stages 2–4 COPD, AUROC in a test set of subjects with GOLD stages 2–4 COPD was 0.83 (for comparison, AUROC in the same test data for model 11 was 0.85, Delong *p*-value = 0.48). Two models did not perform better than model 11. We decided model 11 as our final best-performing model in predicting dyspnea in former and current smokers with and without COPD.

#### Variable Importance

**Fig. 4:**
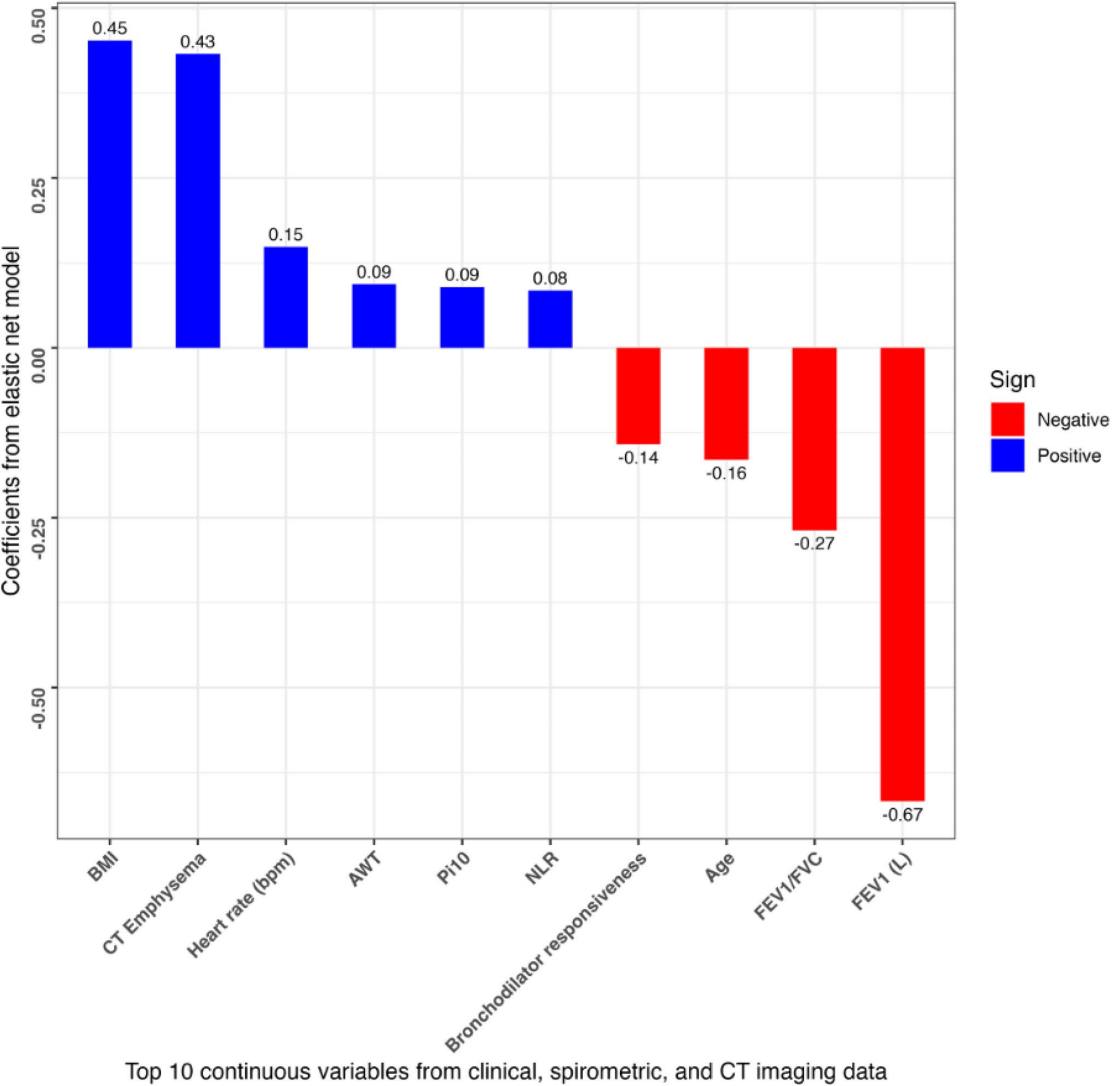

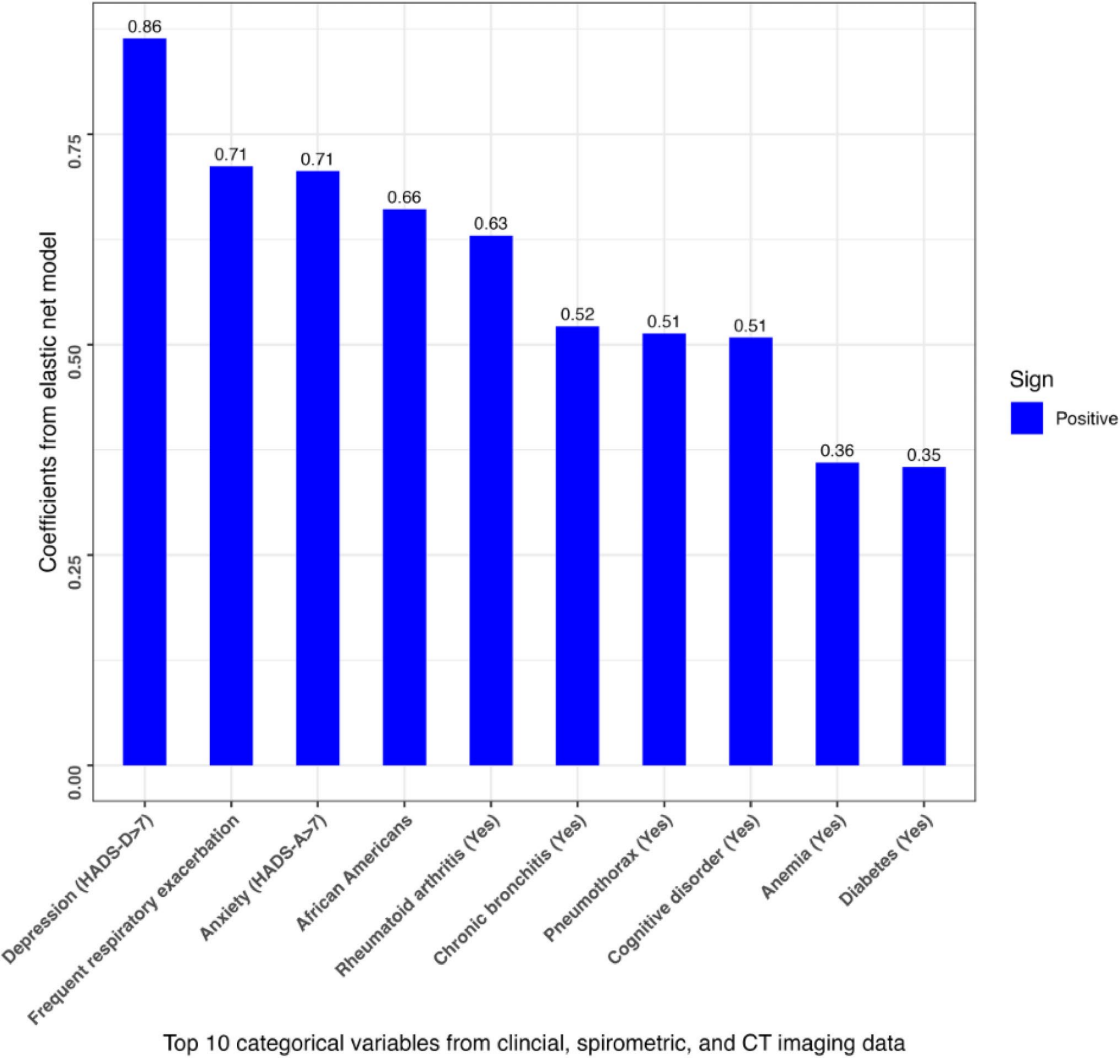
**A** Continuous variable importance scores. The top 10 continuous predictors were selected by the absolute values of their beta coefficients from the elastic net model using clinical, spirometric, and CT imaging variables in COPDGene (Visit 2). The vertical lines represent the magnitude of the coefficient for each feature. All continuous predictors were centered and scaled. Abbreviations: AWT = airway wall thickness; Bronchodilator (BD) responsiveness: post-BD FEV1 (Liters)—pre-BD FEV1 / pre-BD FEV1; BMI = body mass index; bpm = beats per minute; CT = computed tomography; FEV1 (Liters) = pre-bronchodilator forced expiratory volume in 1 s (Liters); FEV1/FVC = pre-bronchodilator forced expiratory volume in 1 s/forced vital capacity; NLR = neutrophil-to-lymphocyte ratio; Pi10 = square root of airway wall area of hypothetical airway with internal perimeter of 10 mm. **B** Categorical variable importance scores. The top 10 categorical predictors were selected by the absolute values of their beta coefficients from the elastic net model using clinical, spirometric, and CT imaging variables in COPDGene (Visit 2). The vertical lines represent the magnitude of the coefficient for each feature. All predictors were centered and scaled. Abbreviations: CT = computed tomography; HADS-A = hospital anxiety and depression scale-anxiety; HADS-D = hospital anxiety and depression scale-depression

The top 10 continuous and categorical predictors were selected based on the absolute values of their beta coefficients from the elastic net model (Model 11), which utilized clinical history, spirometry, and CT imaging in the COPDGene study. To make these more comparable, all continuous variables were mean-centered and scaled before model building. The importance scores for the top ten continuous variables are shown in Fig. 4A. For continuous variables, the factors with the largest importance scores were pre-bronchodilator FEV1 (L) (− 0.67), body mass index (BMI) (0.45), and CT-quantified emphysema (0.43). For categorical variables (Fig. 4B), the most important were the presence of depression (Hospital Anxiety and Depression Scale-Depression (HADS-D) > 7 [15]) (0.86), frequent respiratory exacerbation (0.71), and the presence of anxiety (HADS-Anxiety (HADS-A) > 7 [15]) (0.71).

#### Independent Replication of the Dyspnea Model

Independent validation was performed using the ECLIPSE Study, which enrolled smokers enriched for COPD and obtained extensive clinical history, spirometry, and chest CT characterization. Since there was some difference in variable availability between the two studies, we trained a new model to predict dyspnea using variables present in both COPDGene and ECLIPSE. The characteristics of subjects used for this analysis are shown in Supplemental Table 5. The AUROC for this model was 0.84 in the COPDGene testing dataset and 0.80 in ECLIPSE (Supplemental Figure 2).

## Discussion

Using two large cohorts enriched for smokers with COPD, we developed and validated prediction models for dyspnea. The best-performing model accurately predicts dyspnea in smokers with and without spirometry-driven COPD used by GOLD, achieving an AUROC of 0.85 in the test dataset and 0.80 in the external dataset. We identified novel interactions among multiple risk factors on spirometry-driven COPD severity, as defined by GOLD.

Our study suggests that CT emphysema has a more substantial effect on dyspnea in subjects with PRISM and GOLD stages 2–4 COPD than in smokers with normal spirometry. This finding indicates that abnormal lung structure (emphysema) and diminished lung function (airway obstruction) may drive dyspnea more in clinically diagnosed COPD. Regarding the interaction effects of NLR on COPD severity, evidence suggests that tissue injury in the lungs of smokers with COPD releases inflammatory mediators, activating bronchopulmonary C-fibers and inducing dyspnea [23–25]. Since increased NLR indicates systemic inflammation, it may reflect the “spill-over” of inflammatory mediators in the lung [26] and suggest a link between NLR (and systemic inflammation) and symptom burden in GOLD stages 2–4 COPD.

Chronic bronchitis (chronic cough and phlegm) and frequent respiratory exacerbations are common in smokers with COPD but can also affect those without airway obstruction [27–29]. This suggests that a comprehensive assessment of dyspnea, chronic cough, phlegm, and history of exacerbations may help identify non-COPD smokers at higher risk of dyspnea.

To our knowledge, this is the first predictive model specifically developed to predict dyspnea in smokers with COPD. Our model revealed common and distinct predictors of dyspnea with Olsson et al. [2] Common features included higher BMI, lower spirometric lung function, elevated systemic inflammatory markers, and chronic bronchitis (or cough). Distinct features in our model were CT-quantified emphysema, airway wall thickness, depression, and anxiety. These differences may reflect varying causes of dyspnea in smokers vs. non-smokers, and those with and without COPD.

One challenge in predicting dyspnea is that spirometry and chest CT are not always available. Accordingly, we evaluated how well various combinations of clinical history, spirometry, and chest CT imaging data can predict dyspnea. First, we found that it is possible to obtain accurate dyspnea prediction from standard demographic information combined with a thorough catalog of comorbidities, as observed from the performance of the “extensive clinical variables” model. Second, dyspnea can be predicted with reasonable accuracy from either chest qCT data or spirometry, combined with basic demographic information. Both observations suggest that these models have the potential to identify dyspnea from administrative medical data or as an integrated part of spirometry or chest CT evaluation.

This study has limitations. Our dyspnea models, developed via a cross-sectional design, can’t establish causality. While the model was validated in an independent cohort, demonstrating validity and generalizability, additional validation is needed before clinical use because we did not test it in never-smokers, younger or older populations, or other ethnic groups. In addition, the American Thoracic Society Official Statement highlights that dyspnea assessment is best performed using a multidimensional approach that considers sensory-perceptual, affective distress, and impact domains [30]. We acknowledge that the mMRC dyspnea scale provides a useful but incomplete grading of dyspnea, which may introduce selection bias into the dyspnea prediction model. We focused on mMRC dyspnea measures due to their ease of use and widespread availability.

In summary, interaction models indicated that while the effects of chronic bronchitis and respiratory exacerbations are greater on dyspnea in smokers with preserved spirometry than in those with COPD, the impacts of CT emphysema and systemic inflammation are more significant in smokers with GOLD 2–4 COPD. We present an elastic net model that accurately predicts dyspnea presence using clinical data, spirometry, and/or qCT imaging measures. The most accurate prediction of dyspnea required data from at least two of the three examined domains: demographics/comorbidity, spirometry, or qCT imaging.

## Supplementary Information

Below is the link to the electronic supplementary material.

Supplementary Material 1

Supplementary Material 2

Supplementary Material 3

Supplementary Material 4

Supplementary Material 5

Supplementary Material 6

## Data Availability

The data for this study are accessible via NCBI dbGAP (Accession number pht002239.v4.p2).
