# Supplementary material for: Clinical History, Spirometry, and CT Features Can Predict Dyspnea in Smokers with and without Spirometry-Defined COPD
Source: Lung. 2026 Feb 19;204(1):10. doi: 10.1007/s00408-026-00871-5 (PMC12920348; doi:10.1007/s00408-026-00871-5)
Supplement: Supplementary file 1 — Supplementary Material 1 [file 408_2026_871_MOESM1_ESM.pdf]

Supplemental Table 1. 42 variables included in the prediction algorithms

Clinical history (N = 34)
• *Age<br>• *Sex<br>• *Race<br>• Body mass index (kg/m^2)<br>• Smoking status (Former/current)<br>• Smoking pack-year history<br>• Frequent respiratory exacerbation (Respiratory exacerbation > 2 per year)<br>• Heart rate (bpm)<br>• Pneumothorax (Self-report)<br>• Congestive heart failure (Self-report)<br>• Diabetes (Self-report)<br>• Hypertension (Self-report)<br>• Hyperlipidemia (Self-report)<br>• Pulmonary embolism or Deep vein thrombosis (Self-report)<br>• Peripheral vascular disease (Self-report)<br>• Vertebral compression fracture (Self-report)<br>• Hip fracture (Self-report)<br>• Osteoarthritis (Self-report)<br>• Osteoporosis (Self-report)<br>• Rheumatoid arthritis (Self-report)<br>• Chronic bronchitis (Chronic cough and phlegm for at least 3 months a year for at least 2 consecutive years)<br>• Cognitive disorder (Self-report)<br>• Anemia (Self-report)<br>• Kidney disease (Self-report)<br>• Liver disease (Self-report)<br>• Lung cancer (Self-report)<br>• Cardiovascular disease (A composite of self-reported diagnoses of angina, coronary artery disease, heart attack, or atrial fibrillation, or self-reported history of coronary artery bypass grafting or angioplasty)<br>• Cerebrovascular disease (A composite of self-reported diagnoses of stroke or transient ischemic aneurysm)<br>• GERD/ulcer (A composite of self-reported diagnoses of gastroesophageal reflux disease or stomach ulcer)<br>• Depression (HADS-D > 7)<br>• Anxiety (HADS-A > 7)<br>• Hemoglobin (g/dL)<br>• Eosinophil (k/uL)<br>• Neutrophil-to-lymphocyte ratio (NLR)
Spirometry (N = 3)
• Pre-bronchodilator FEV1 (L)<br>• Pre-bronchodilator FEV1/FVC, predicted<br>• Pre- and post-bronchodilator FEV1, % change
Chest CT imaging (N = 5)

- CT quantified total emphysema, %LAA-950
- Emphysema distribution (Upper over lower lung third %LAA-950 ratio)
- Pi10 (Square root of the wall area of a hypothetical airway of 10-mm internal perimeter)
- Segmental airway wall thickness (Obtained along the center line of the lumen, in the middle third of the airway segment, for one segmental airway of each lung lobe)
- Expiratory CT-measured estimated mass of the lung (as opposed to the CT scan obtained after full inspiration)

* The basic clinical model includes age, sex, and race, while the extensive clinical model incorporates 34 variables from clinical history.

Abbreviations: BMI = body mass index; bpm = beats per minute; COPD = chronic obstructive pulmonary disease; CT = computed tomography; FEV1 = forced expiratory volume in 1 second; FVC = forced vital capacity; GERD = gastroesophageal reflux disease; Hgb = hemoglobin; HADS-A = hospital anxiety and depression scale – anxiety; HADS-D = hospital anxiety and depression scale – depression; N = number; NLR = neutrophil-to-lymphocyte ratio; NS = not significant; LAA = low attenuation areas; Pi10 = square root of airway wall area of hypothetical airway with internal perimeter of 10 mm
