# Supplementary material for: Clinical History, Spirometry, and CT Features Can Predict Dyspnea in Smokers with and without Spirometry-Defined COPD
Source: Lung. 2026 Feb 19;204(1):10. doi: 10.1007/s00408-026-00871-5 (PMC12920348; doi:10.1007/s00408-026-00871-5)
Supplement: Supplementary file 3 — Supplementary Material 3 [file 408_2026_871_MOESM3_ESM.pdf]

Table 1. Characteristics of 4013 individuals with a history of smoking in the training dataset from COPDGene visit 2

	**No Dyspnea** N = 2,553 (63.6%)*	**Yes Dyspnea** N = 1,460 (36.4%)*	**p-value**†
**mMRC dyspnea score**			<0.001
0 (Dyspnea only with strenuous exercise)	2,047 (80%)	0 (0%)	
1 (Dyspnea when walking up a slight hill)	506 (20%)	0 (0%)	
2 (Walks slower than people of the same age because of dyspnea or must stop for breath when walking at own pace)	0 (0%)	493 (34%)	
3 (Stop for breath after walking 100 yards or after a few minutes)	0 (0%)	674 (46%)	
4 (Too dyspneic to leave house or breathless when dressing)	0 (0%)	293 (20%)	
**GOLD grade**			<0.001
Normal spirometry	1,405 (55%)	361 (25%)	
PRISm	284 (11%)	208 (14%)	
Mild (GOLD 1)	311 (12%)	77 (5.3%)	
Moderate-to-very-severe (GOLD 2-4)	553 (22%)	814 (56%)	
**Frequent respiratory exacerbation**	77 (3.0%)	216 (15%)	<0.001
**Age, yr**	65 (8)	65 (9)	0.9
**Sex, % male**	1,369 (54%)	687 (47%)	<0.001
**Race, % NHW**	1,906 (75%)	917 (63%)	<0.001
**BMI**	28 (6)	30 (7)	<0.001
**Current smoker**	981 (38%)	583 (40%)	0.3
**Smoking pack-years**	41 (22)	49 (25)	<0.001
**Heart rate at rest (bpm)**	72 (12)	76 (13)	<0.001
**Hemoglobin (g/dL)**	14.08 (1.37)	13.77 (1.61)	<0.001
**Eosinophils (K/uL)**	0.18 (0.14)	0.19 (0.15)	0.9
**Neutrophil-to-lymphocyte ratio**	0.69 (0.49)	0.81 (0.60)	<0.001
**Self-reported comorbid conditions**			
Anxiety (HADS-A > 7)	356 (14%)	424 (29%)	<0.001
Depression (HADS-D > 7)	172 (6.7%)	322 (22%)	<0.001
Congestive heart failure	46 (1.8%)	97 (6.6%)	<0.001
Cardiovascular disease	386 (15%)	335 (23%)	<0.001

	No Dyspnea<br>N = 2,553 (63.6%)*	Yes Dyspnea<br>N = 1,460 (36.4%)*	p-value†
Cerebrovascular disease	129 (5.1%)	127 (8.7%)	<0.001
GERD or gastric ulcer	757 (30%)	561 (38%)	<0.001
Diabetes	352 (14%)	360 (25%)	<0.001
Hypertension	1,190 (47%)	870 (60%)	<0.001
Peripheral vascular disease	58 (2.3%)	71 (4.9%)	<0.001
Osteoporosis	239 (9.4%)	179 (12%)	0.004
Chronic bronchitis	254 (9.9%)	341 (23%)	<0.001
Anemia	254 (9.9%)	230 (16%)	<0.001
Kidney disease	60 (2.4%)	66 (4.5%)	<0.001
**Spirometry**			
Pre-bronchodilator FEV1 (L)	2.31 (0.75)	1.59 (0.77)	<0.001
Pre-bronchodilator FEV1/FVC	0.70 (0.11)	0.59 (0.17)	<0.001
FEV1 (% predicted) ‡	87 (20)	63 (25)	<0.001
Pre- and post-bronchodilator FEV1% change	5 (8)	7 (12)	<0.001
**Quantitative CT imaging characteristics**			
CT Emphysema (%LAA-950)	4 (6)	9 (12)	<0.001
Emphysema distribution ratio (Upper over lower lung third %LAA-950 ratio)	0.49 (0.90)	0.51 (0.90)	0.7
Pi10 (mm)	2.13 (0.50)	2.56 (0.60)	<0.001
Segmental airway wall thickening (mm)	1.00 (0.21)	1.10 (0.23)	<0.001
Estimated lung mass on expiratory CT (g)	898 (173)	864 (185)	<0.001

Abbreviations: BMI = body mass index; CT = computed tomography; FEV1 = forced expiratory volume in one second; FVC = forced vital capacity; GERD = Gastroesophageal reflux disease; GOLD = Global Initiative for Chronic Obstructive Lung Disease; HADS-A = hospital anxiety and depression scale – anxiety; HADS-D = hospital anxiety and depression scale – depression; %LAA-950 = percentage of CT pixels with attenuation value less than -950 Hounsfield units; mMRC = modified Medical Research Council dyspnea scale; N = number; NHW = non-Hispanic White; Pi10 = square root of airway wall area of hypothetical airway with internal perimeter of 10 mm; PRISm = preserved ratio impaired spirometry; SD = standard deviation; yr = years. Continuous variables are expressed as mean and standard deviation (SD). Categorical variables are expressed as absolute values and/or percentages. FEV1 (% predicted) is calculated using the GLI global equation (Global Lung Function Initiative) ‡.

* N (%); Mean (SD)

† T-test; Pearson's Chi-squared test; Wilcoxon rank sum test

‡ Quanjer et al., ERS Global Lung Function Initiative. Multi-ethnic reference values for spirometry for the 3-95-yr age range: the global lung function 2012 equations. Eur Respir J. 2012 Dec;40(6):1324-43.
