# Supplementary material for: Clinical History, Spirometry, and CT Features Can Predict Dyspnea in Smokers with and without Spirometry-Defined COPD
Source: Lung. 2026 Feb 19;204(1):10. doi: 10.1007/s00408-026-00871-5 (PMC12920348; doi:10.1007/s00408-026-00871-5)
Supplement: Supplementary file 4 — Supplementary Material 4 [file 408_2026_871_MOESM4_ESM.pdf]

Supplemental Table 3. Bivariable and multivariable logistic regression models of the relationship between dyspnea and clinical history, spirometry, and chest CT imaging characteristics in 4013 former and current smokers with and without COPD in the COPDGene training dataset

	**Bivariable models**	**Multivariable models**[§]
	**Beta (SE)**	**Beta (SE)**
COPD stage (Reference: Normal spirometry)	--	--
PRISm	1.05 (0.11) **	--
Mild (GOLD stage 1)	-0.04(0.14) [ns]	--
Moderate to very severe (GOLD stage 2-4)	1.75 (0.08) **	--
Pre-bronchodilator FEV1(L)	-1.06 (0.04) **	--
Pre-bronchodilator FEV1/FVC ratio	-0.79 (0.04) **	--
Age, yr	0.01 (0.03) [ns]	--
Sex, female (Reference: Male)	0.26 (0.07) **	--
Race, Black or African American (Reference: NHW)	0.56 (0.07) **	--
Frequent respiratory exacerbation (Reference: No)	1.72 (0.14) **	1.30 (0.15) **
Bronchodilator responsiveness (% FEV1 pre vs. post)	0.20 (0.03) **	0.06 (0.04) [ns]
BMI	0.32 (0.03) **	0.41 (0.04) **
Smoking status (Reference: Former smoker)	0.06 (0.07) [ns]	-0.14(0.08) [ns]
Smoking pack-years	0.33 (0.03) **	0.27 (0.04) **
Heart rate, bpm	0.38 (0.03) **	0.28 (0.04) **
Hgb (g/dL)	-0.21(0.03) **	-0.14 (0.04) **
Eosinophils (k/uL)	0.02 (0.03) [ns]	0.03 (0.04) [ns]
NLR	0.22 (0.03) **	0.26 (0.04) **
Pneumothorax (Reference: No)	1.02 (0.20) **	0.86 (0.22) **
Congestive heart failure (Reference: No)	1.36 (0.18) **	1.02 (0.20) **
Diabetes (Reference: No)	0.72 (0.08) **	0.68 (0.09) **
Hypertension (Reference: No)	0.52 (0.07) **	0.35 (0.07) **
Hyperlipidemia (Reference: No)	0.13 (0.07) *	0.20 (0.07) *
Pulmonary embolism or Deep vein thrombosis (Reference: No)	0.56 (0.14) **	0.63 (0.16) **
Peripheral vascular disease (Reference: No)	0.79 (0.18) **	0.74 (0.20) **
Vertebral compression fracture (Reference: No)	0.31 (0.15) *	0.24 (0.16) [ns]
Hip fracture (Reference: No)	0.46 (0.21) *	0.41 (0.23) [ns]
Osteoarthritis (Reference: No)	0.37 (0.07) **	0.49 (0.08) **
Osteoporosis (Reference: No)	0.30 (0.10) **	0.06 (0.12) [ns]
Rheumatoid arthritis (Reference: No)	0.70 (0.13) **	0.80 (0.15) **
Chronic bronchitis (Reference: No)	1.01 (0.09) **	0.82 (0.10) **
Cognitive disorder (Reference: No)	0.88 (0.22) **	0.88 (0.24) **
Anemia (Reference: No)	0.53 (0.10) **	0.59 (0.11) **

Kidney disease (Reference: No)	0.68 (0.18) **	0.79 (0.20) **
Liver disease (Reference: No)	0.37 (0.15) *	0.35 (0.16) *
Lung cancer (Reference: No)	0.97 (0.37) **	0.53 (0.41) ns
Cardiovascular disease (Reference: No)	0.51 (0.08) **	0.54 (0.10) **
Cerebrovascular disease (Reference: No)	0.58 (0.13) **	0.59 (0.14) **
Gastrointestinal disease (Reference: No)	0.39 (0.07) **	0.38 (0.08) **
Anxiety (Reference: HADS-A ≤ 7)	0.93 (0.08) **	1.00 (0.09) **
Depression (Reference: HADS-D ≤ 7)	1.37 (0.10) **	1.37 (0.11) **
CT Emphysema (LAA%950)	0.68 (0.04) **	0.49 (0.05) **
Pi10 (mm)	0.81 (0.04) **	0.45 (0.04) **
Segmental airway wall thickening (mm)	0.48 (0.03) **	0.39 (0.04) **
Estimated lung mass on expiratory CT (g)	-0.20 (0.03) **	0.03 (0.05) ns
Emphysema distribution (upper over lower lung third % LAA-950 ratio)	0.02 (0.03) ns	0.02 (0.04) ns

Abbreviations: BMI = body mass index; bpm = beats per minute; COPD = chronic obstructive pulmonary disease; CT = computed tomography; FEV1 = forced expiratory volume in 1 second; FVC = functional volume capacity; g/dL = grams per deciliter; Hgb = hemoglobin; HADS-A = hospital anxiety and depression scale – anxiety; HADS-D = hospital anxiety and depression scale – depression; k/uL = thousands per microliter; NHW = non-Hispanic White; NLR = neutrophil-to-lymphocyte ratio; LAA-950 (% of CT voxels below -950HU) = low attenuation areas; Pi10 = square root of airway wall area of hypothetical airway with internal perimeter of 10 mm; PRISm = preserved ratio impaired spirometry; SE = standard error; yr = years;

NS not significant; * p<.05; ** p<.005

Each row corresponds to separate bivariate and multivariate models for the presence of dyspnea, defined by mMRC ≥ 2.

§ Each multivariate model is adjusted for GOLD spirometric stage, age, sex, and race. The variable "GOLD spirometric stage" was coded into four groups for analysis: normal spirometry (GOLD stage 0), preserved ratio impaired spirometry (PRISm), mild COPD (GOLD stage 1), and moderate to severe COPD (GOLD stages 2-4).
