# Supplementary material for: Clinical History, Spirometry, and CT Features Can Predict Dyspnea in Smokers with and without Spirometry-Defined COPD
Source: Lung. 2026 Feb 19;204(1):10. doi: 10.1007/s00408-026-00871-5 (PMC12920348; doi:10.1007/s00408-026-00871-5)
Supplement: Supplementary file 5 — Supplementary Material 5 [file 408_2026_871_MOESM5_ESM.pdf]

Supplemental Table 4. Characteristics of subjects in the new models of the COPDGene training and test dataset at Visit 2

	**COPDGene*** Train dataset (N = 4060)	**COPDGene*** Test dataset (N = 1015)	**ECLIPSE*** (N = 2290)
**Age, yr**	65 (9)	65 (9)	61.4 (8.3)
**Sex, % male**	2,073 (51%)	492 (48%)	1386 (60.5%)
**Race, % NHW**	2,853 (70%)	693 (68%)	2236 (97.6%)
**BMI**	29 (6)	29 (6)	26.5 (5.4)
**Current smoker**	1,565 (39%)	424 (42%)	635 (27.7%)
**Smoking pack-years**	43 (23)	45 (26)	41.6 (28.4)
**Heart rate (bpm)**	73 (12)	73 (12)	76.3 (13.2)
**Eosinophils (K/uL)**	0.2 (0.2)	0.2 (0.1)	0.2 (0.2)
**Neutrophil-to-lymphocyte ratio**	0.7 (0.5)	0.7 (0.5)	1.3 (0.7)
**Spirometry**			
Pre-bronchodilator FEV1(L)	2.1 (0.8)	2.0 (0.8)	1.7 (1.0)
Pre-bronchodilator FEV1/FVC	0.7 (0.1)	0.7 (0.1)	51.4 (17.2)
Pre- and post-bronchodilator FEV1% change	6 (10)	6 (10)	9.3 (12.6)
**Frequent respiratory exacerbation**	277 (6.8%)	76 (7.5%)	178 (7.8%)
**Self-reported comorbid conditions**			
Anxiety (HADS-A>7)	798 (20%)	191 (19%)	339 (14.8%)
Depression (HADS-D>7)	525 (13%)	124 (12%)	369 (16.1%)
Congestive heart failure	146 (3.6%)	26 (2.6%)	99 (4.3%)

	**COPDGene***<br>Train dataset (N = 4060)	**COPDGene***<br>Test dataset (N = 1015)	**ECLIPSE***<br>(N = 2290)
Cardiovascular disease	707 (17%)	180 (18%)	200 (8.7%)
Cerebrovascular disease	254 (6.3%)	71 (7.0%)	75 (3.3%)
GERD or gastric ulcer	1,321 (33%)	332 (33%)	577 (25.2%)
Diabetes	703 (17%)	186 (18%)	14 (0.6%)
Hypertension	2,070 (51%)	527 (52%)	791 (34.5%)
Osteoporosis	410 (10%)	112 (11%)	249 (10.9%)
Chronic bronchitis	624 (15%)	136 (13%)	619 (27.0%)
**CT imaging characteristics**			
CT Emphysema (%LAA-950)	6 (9)	5 (9)	14.6 (12.6)
Pi10 (mm)	2.3 (0.6)	2.3 (0.6)	4.4 (0.2)
Segmental airway wall thickening (mm for the COPDGENE, % for the ECLIPSE)	1.04 (0.2)	1.03 (0.2)	65.5 (3.8)

Abbreviations: BMI = body mass index; CT = computed tomography; FEV1 = forced expiratory volume in one second; FVC = forced vital capacity; GERD = Gastroesophageal reflux disease; HADS-A = hospital anxiety and depression scale – anxiety; HADS-D = hospital anxiety and depression scale – depression; %LAA-950 = percentage of CT pixels with attenuation value less than -950 Hounsfield units; N = number; NHW = non-Hispanic White; Pi10 = square root of airway wall area of hypothetical airway with internal perimeter of 10 mm; SD = standard deviation; yr = years.

* Continuous variables are expressed as mean and standard deviation. Categorical variables are expressed as absolute values (N) and percentages.
