# Supplementary material for: Clinical History, Spirometry, and CT Features Can Predict Dyspnea in Smokers with and without Spirometry-Defined COPD
Source: Lung. 2026 Feb 19;204(1):10. doi: 10.1007/s00408-026-00871-5 (PMC12920348; doi:10.1007/s00408-026-00871-5)
Supplement: Supplementary file 6 — Supplementary Material 6 [file 408_2026_871_MOESM6_ESM.docx]

**
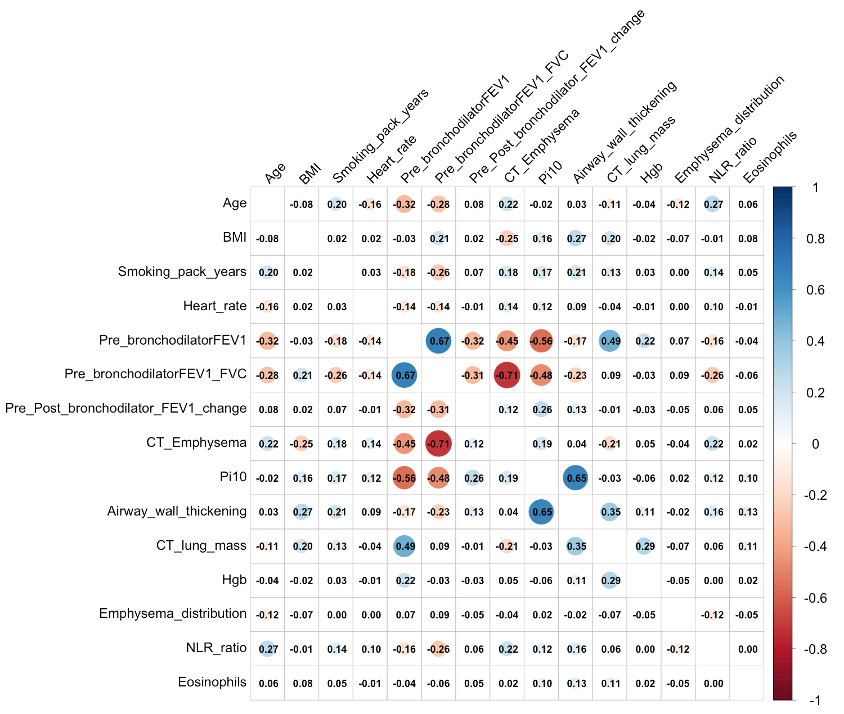
**

**Supplemental Figure 1(A). Correlation matrix of continuous variables.** The matrix displays the correlation between pairs of continuous variables, with the color intensity and correlation coefficient indicating the strength and direction of the relationship. The color gradient ranges from negative (-1, representing a strong negative correlation) to positive (+1, representing a strong direct correlation), as shown in the color scale on the right. Smaller circles with lighter colors indicate a weaker correlation. Correlation coefficients are also provided within the individual matrix cells.

**
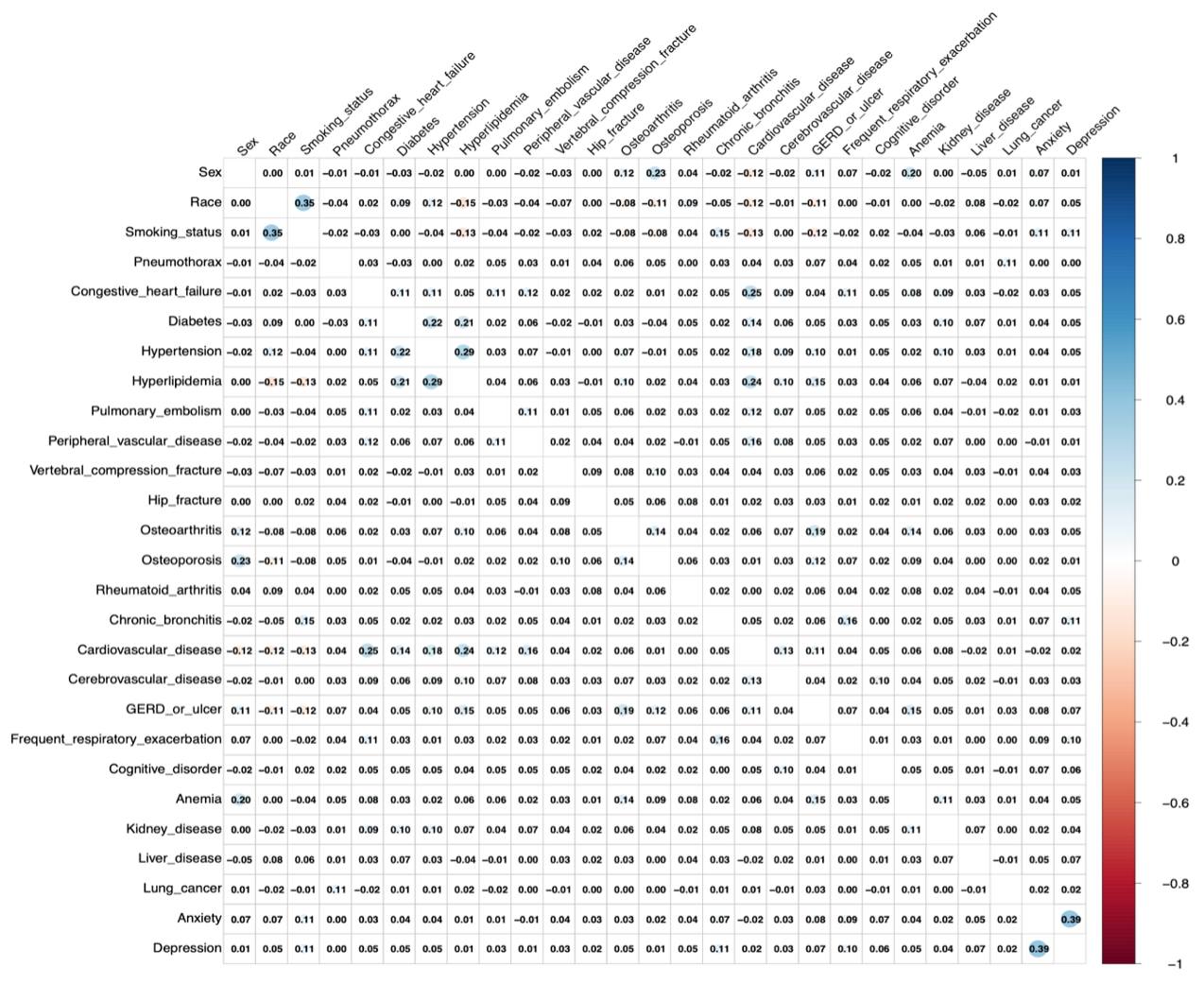
**

**Supplemental Figure 1(B). Correlation matrix of categorical variables.** The matrix displays Kendall’s rank correlation between pairs of continuous variables, with the color intensity and correlation coefficient indicating the strength and direction of the relationship. The color gradient ranges from negative (-1, representing a strong negative correlation) to positive (+1, representing a strong direct correlation), as shown in the color scale on the right. Smaller circles with lighter colors indicate a weaker correlation. Correlation coefficients are also provided within the individual matrix cells.

**
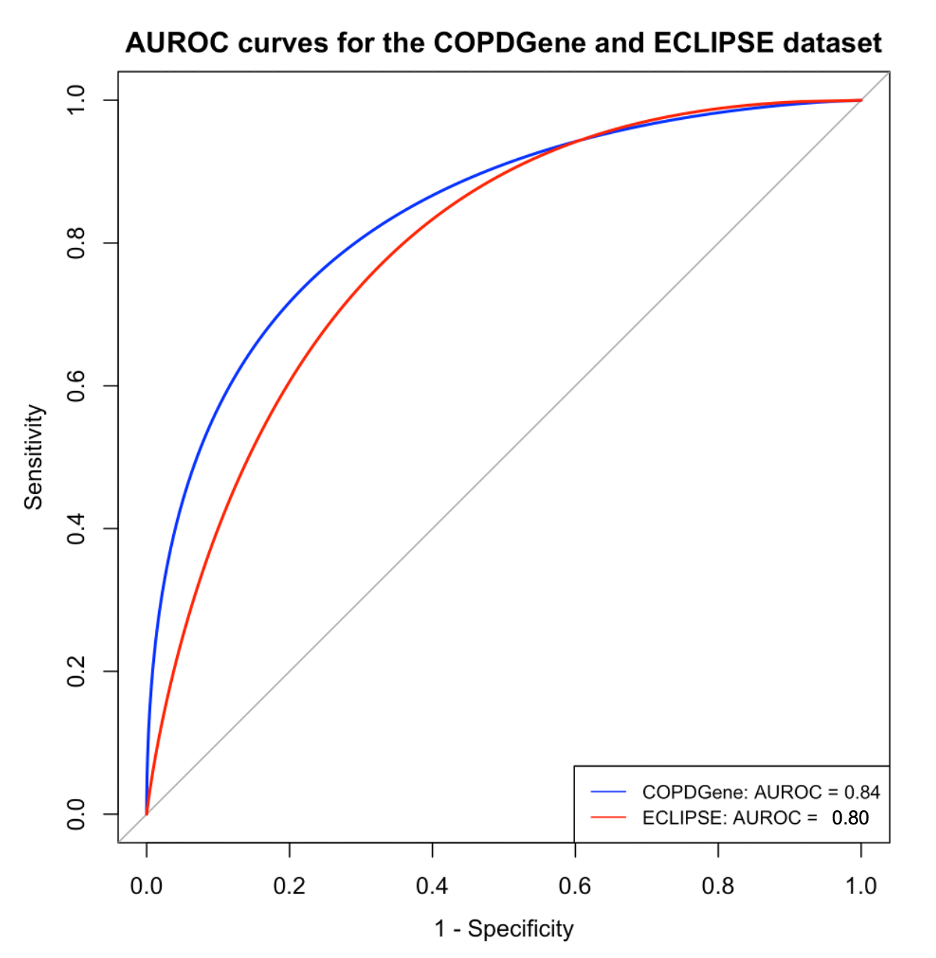
**

**Supplemental Figure 2. AUROC curves of the models predicting dyspnea in the test dataset of the COPDGene study (Visit 2) and the external dataset of the ECLIPSE study.** AUROC = area under the receiver operating characteristics; ECLIPSE = Evaluation of COPD Longitudinally to Identify Predictive Surrogate Endpoints; COPD = chronic obstructive pulmonary disease
